# Use of simulation to improve nursing students’ medication administration competence: a mixed-method study

**DOI:** 10.1186/s12912-022-00897-z

**Published:** 2022-05-16

**Authors:** Pol-Castañeda Sandra, Carrero-Planells Alba, Moreno-Mulet Cristina

**Affiliations:** 1 Department of Nursing and Physiotherapy, University of the Balearic Islands, Palma, Balearic Islands, Spain; 2 Care, Chronicity and Health Evidences Research Group, Health Research Institute of the Balearic Islands (IdISBa), 07010 Palma, Spain

**Keywords:** Simulation, Medication administration, Medication therapy management, Nursing education, Patient safety, Mix-methods

## Abstract

**Background:**

Medication administration errors are among the most important adverse events in healthcare systems. To minimise the risk of this occurring, nursing training programmes should emphasise the overriding priority of patient safety. In this respect, simulation can be a valuable resource in teaching procedures, for patient safety in general and safe medication administration in particular. In this study, we evaluate the use of a simulation-based activity for students to acquire skills in safe medication administration, and consider the students’ perceptions of this activity.

**Methods:**

Second-year nursing students enrolled in the subject of pharmacology at a Spanish university during the academic year 2018–2019 were invited to participate in this mixed-method study. Their acquisition of professional competencies via a simulation exercise was evaluated according to the ‘six rights’. Before the simulation, each student completed a researcher-developed online questionnaire. The simulation was evaluated by the students’ tutor, using a checklist. A descriptive analysis was made of the data obtained from the questionnaire and during the simulation. At the end of the semester, the students' opinions were recorded in the questionnaire, in response to an open question. A content analysis was made of the responses to the open question.

**Results:**

The simulation exercise was performed by 179 students, of whom 73 had previously completed the questionnaire. Analysis showed that, in comparison with the pre-simulation questionnaire results, compliance with the six rights improved in all dimensions except data documentation: right patient (from 64.4% to 83.3%); right medication (from 60.3% to 95.8%); right dose (from 60.3% to 100%); right route (from 54.8% to 95.8%); right time (from 24.7% to 70.8%); the right documentation result fell from 54.8% to 45.8%. The students expressed their satisfaction with the simulation method, affirming that it brought them closer to the reality of health care.

**Conclusions:**

Simulation is a useful tool for the acquisition of skills in medication administration. The students were satisfied with the simulation capacity to bridge the gap between theory and practice. Moreover, simulation represents an added teaching resource in the nursing degree curriculum and is expected to enhance patient safety.

## Background

According to the World Health Organization (WHO), unsafe medication practices and medication errors are a leading cause of injury and avoidable harm in health care systems across the world [1, 2]. These errors contribute to patient morbidity and mortality and provoke annual financial costs of around $42 million a year. The World Health Organization (WHO) has set the target of reducing severe, avoidable medication-related harm by 50% during 2022 [3]. Among medication errors, those of administration are the most common [4] and nurses can and should play a fundamental role in their prevention [4–6]. The administration of medication is a basic nursing skill and it is nurses’ responsibility to perform it safely and effectively [7].

Patient safety should be a central aspect of nursing skills training programmes [8, 9]. In this respect, the WHO Patient Safety Curriculum Guide [10] urges nursing schools to instil the need to prioritise patient safety before students become healthcare professionals, with particular emphasis on ensuring safe medication management [11–13]. In Spain, Cervera-Gasch [14] and Mira [15, 16] refer to the need to improve training in patient safety in nursing studies after having evaluated the knowledge and attitudes of the students. The National Strategy for Patient Safety of the National Health System (2015–2020) proposes promoting basic training in patient safety for all healthcare professionals as one of its objectives. Among its recommendations, it includes the need to agree on a minimum curriculum for basic training in patient safety in undergraduate and postgraduate studies [17]. However, it does not develop teaching implementation strategies or methodologies to carry it out effectively [14, 15]. The WHO [10] recommends combining various teaching methods and formats, and the simulation approach is currently attracting much international attention.

Simulation is an active learning tool that, through guided experiences, aims to reproduce reality in a controlled, interactive context, addressing important aspects of real practice in a risk-free environment [18]. Designing an effective simulation scenario requires careful planning and it is is essential to ensure the achievement of teaching goals. Scenario design must also include consideration of the level of fidelity, which refers to the degree of realism in which the student is immersed. All simulation scenarios should be designed to address a perceived knowledge or performance gap [19]. Simulation has been shown to be more effective than many traditional teaching techniques and it is being implemented in universities worldwide [20–23]. Studies have analysed the use of simulation as a strategy for the acquisition of confidence and skills in various areas of clinical safety, including the administration of medication and the prevention of adverse events related to this administration, with favourable results [7, 9, 12, 24–32].

In Spain, unlike other countries where the administration of medication is a skill that nurses gradually acquire in their working experience, newly-graduated nurses are expected to be able to administer any type of medication, as and when required. Therefore, appropriate training, before they initiate professional activity, is vital to ensure that nurses have this essential competency. The acquisition of this competence is regulated by the ORDER CIN / 2134/2008, of July 3, that determines the basic contents of the nursing curriculum, including those related to pharmacology [33].

At the University of the Balearic Islands (Spain), medication administration competency is acquired through theoretical classes, the most specific of which is pharmacology (taught in the third semester) and through clinical practice classes (in the fourth and subsequent semesters). In these practice classes, students perform activities based on low-fidelity simulation to prepare them for real-world healthcare experience. However, we don’t have previous experiences in the use of high-fidelity simulation with which to work on these aspects.

This study was conducted to evaluate the implementation of a teaching innovation project. The primary aim was to evaluate nursing students’ acquisition of skills in the safe administration of medication, using a simulation-based activity (SBA). The secondary aim was to ascertain the students’ overall opinion of the activity.

## Methods

### Design

The study consisted of a mixed-methods design. The mixed methods approach can be seen as offering a third paradigm for social research through the way it combines quantitative and qualitative methodologies [34]. This methodology will enable us to seek a more panoramic view of our innovation project, viewing phenomena from different viewpoints and through diverse research lenses [35, 36].

### Setting and participants

The students involved were in the second year of a nursing degree course at the University of Balearic Islands, enrolled in the subject of pharmacology during the academic year 2018–2019. No exclusion criteria were established. Convenience sampling was performed [37].

At the beginning of the semester, the professor responsible for the pharmacology subject -and the main researcher of the project- verbally explained to the students the realization of this teaching innovation project. In addition, a space was set up on the virtual platform of the subject with the relevant information (project aims, anonimation and use of data, voluntary participation, and no impact on the evaluation of the subject) so that the student could decide if they voluntary wanted to participate in the project. The students were able to clarify any questions with the teacher.

### Simulation-based activity

The SBA was designed following the recommendations of the INACSL Standards of Best Practice: Simulation Design [38]. Taking into account the students’ lack of experience in these areas, three simple scenarios were presented to teach knowledge skills (medication administration as a process) and non-technical skills (safety, communication, confidentiality, etc.). Each of the cases presented involved a hospital patient to whom endovenous medication should be administered [39] (Table 1). We contemplate the teaching of the medication administration process and its evaluation under to the ‘six rights’ framework: right patient, right drug, right time, right route, right dose and right documentation [4, 40, 41].

**Table 1:** Clinical scenarios for the SBA

**Scenario**	**Scenario 1:** 72-year-old woman attended in A&E for vomiting, diarrhoea and muscle cramps, diagnosed with hypokalaemia	**Scenario 2:** 80-year-old man admitted for a respiratory infection; body weight 60 kg	**Scenario 3:** 44-year-old woman admitted for paracentesis of 5 L of ascitic fluid
Medication regimen	500 ml PS + 30 mEq KCl every 12 h, IV (continuous infusion)	Ceftazidime 100 mg / kg / day. Distribute in 3 administration, every 8 h	Human albumin 6 g per litre of ascitic fluid obtained (intermittent infusion). Dilute in 250 ml of physiological serum and administer for 40 min
Drug presentation	10 ml ampoule of 2 M KCl	2 g vial of ceftazidime powder	50 ml vial of 20% human albumin
Intervention	Calculate the quantity (ml) of KCl to be added to 500 ml of PS Calculate how many ampoules of KCl are needed Calculate the infusion rate of the medication (ml/h)	Calculate the quantity (g) of ceftazidime to be diluted in 250 ml of PS Calculate how many vials of ceftazidime are needed for each infusion Calculate the infusion rate of the medication (ml/h)	Calculate the quantity (ml) of human albumin to be administered Calculate how many vials of human albumin are needed Calculate the infusion rate (ml/h)

*Abbreviations*: *KCl* Potassium chloride, *PS* Physiological serum

Two weeks before the simulation activity, a prebriefing session was conducted by the subject teacher trained in clinical simulation who carried out the SBA, in which the students were given the case files. These files were prepared for each scenario, including images of the materials to be used and a description of the evaluation method that would be applied. In this prebriefing session, the students were instructed to plan the procedure to be adopted and to clarify any questions they might have. The simulation scenarios were conducted in 24 groups of 6–8 students, each of whom played a different role (nurse, patient, caregiver, student observer, etc.). Before starting, the teacher reminded the students of the aims pursued and contextualised the exercise, in a clinical setting. The students had time to distribute the roles and to design strategies to verify the six rights. During the 15-min scenario, the student who played the nurse rol simulated the correct administration of medication to the patient, including all the steps of the process (preparation, administration and assessment). To consider valid the verification of the right, the check had to be carried out by this student. The rest of the students, from their assigned role, could give clues to guide the nurse. The instructions for the teacher included details on the preparation of the simulated situation and the possibility of providing some degree of assistance, such as “voiceover” during the activity. After the simulation had concluded, there was a 5-min debriefing session to consolidate the learning process, in which a critical analysis was made of the situation, with particular attention to the students’ fulfilment (or otherwise) of the six rights. At the end of the session, when all groups (2–5 groups every session) had completed the SBA, a discussion was carried out with the same debriefing objectives for an approximate duration of 20 min.

### Data collection

The students’ acquisition of skills in the safe administration of medication was assessed by means of a prospective, descriptive study of a SBA. In this activity, each student first completed a researcher-developed questionnaire (Pre-S questionnaire, Table 2), which presented a clinical case similar to the one used in the simulation. The clinical case proposed the administration of medication to two hospitalised patients. Patient A was a man with heart disease and allergic to penicillin. Patient B was a man in severe pain who required a dose of morphine. The questionnaire was designed to assess the students’ knowledge of the six rights, with eight questions (multiple choice and open). Participation was voluntary. The questionnaire was completed on-line, and a time of 40 min was allowed for this purpose. The students’ performance of the SBA was then assessed by their tutor, on a purpose-designed evaluation form (checklist), based on the Medication Administration Safety Assessment Tool (MASAT) [40], consisting of eight dichotomous items regarding compliance or otherwise with the six rights (Table 2). This researcher-developed approach was adopted because to date no gold standard instrument has been established to evaluate this type of process. This absence of generalised application is mainly due to the fact that such evaluations are affected by subjective experience, perceptions, training and the evaluator’s own knowledge [7].

**Table 2:** Evaluation of Pre-S questionnaire and SBA

	**Pre-S questionnaire**	**Expected interventions in the SBA**
Before medication administration	Which of the following drugs poses a serious risk if administered to patient A? (*multi-choice question*)	Check for allergies, susceptibilities, risks, health status, diagnosis according to nurses and doctors, expected results, possible adverse effects Before administration, prepare medication and perform proper hand washing. *(Yes/No)*
Right patient	How would you identify the patient? (*open question*)	At least, check the patient's name against the ID bracelet. (*Yes/No*)
Right medication	Which of the following drugs cannot be administered to the patient A? (*Two multi-choice questions*) Just before preparing the drug dilution (morphic chloride), what actions should you take to verify that the drug presentation is safe? (*Open question*)	Select the drug to be administered from different drugs and presentations. (*Yes/No*)
Right dose	Patient B is prescribed 5 mg of morphic chloride (1 ml 2% ampoules). How many ml of the morphine ampoule are needed? *(Multiple choice question*)	Correctly calculate the dose to be administered (*Yes/No*)
Right route	The morphine that you have just prepared should not be administered by which of these routes? (*Multiple choice question*)	Select appropriate material for drug administration and simulate administration by the appropriate route. (*Yes/No*)
Right time	An unforeseen event occurs and you must leave the room. Which of the drugs that you must administer to patient A and/or patient B can be delayed for 30 min? (*Multiple choice question*)	Explicitly show that the time at which the medication must be administered has been checked (*Yes/No*)
Right documentation	Regarding the administration of morphine to patient B, what relevant information should you record in the medical history? (*Open question*)	On concluding drug administration, record this fact, including the dose and any incident occurring during the administration. (*Yes/No*)
After medication administration	-	Monitor the treatment effects, side effects and possible interactions. (*Yes/No*)

At the end of the first semester, the students were asked (in an open question) for their opinions about the SBA.

The study data were compiled from November 2018 to February 2019.

### Data analysis

We performed a descriptive analysis of the study population and the results of the Pre-S questionnaire and the SBA data. The objective was to compare the data through tables of frequencies and percentages to know the evolution of the students in the acquisition of skills in the safe medication administration. The statistical software used to analyze the data was SPSS v22.0.

An inductive content analysis was made of the answers given to the open question on perceptions of the SBA [42]. Answers were codified independently by three researchers. Once finished, all researchers met to compare their results. Through a process of dialogue and comparison, they reached an agreement on the coding system. Once the list of codes had been completed, the researchers drew up the analysis categories and revised the codes under each of them. Manual analysis was performed. One of the researchers – nursing teacher who carried out the SBA – helped interpret the educational context, specially the students’ opinions expressed during the debriefing.

### Methods of rigor

The SBA has been designed according to the recommendations of the INACSL Standards. The variables collected during the simulation were measured through direct observation by a single instructor. In relation to the qualitative analysis of the students' opinion, the triangulation of the three researchers stands out as a method of rigor and the reflexivity of one of them who facilitated the understanding of the context of the SBA.

### Ethical issues

The processing, communication, and transfer of the personal data belonging to all participants were carried out in compliance with the Declaration of Helsinki and the provisions of Spanish Organic Law 3/2018. The study was approved by the Institute for Educational Research and Innovation of the University of the Balearic Islands (PID 181,957) -according to the regulations of our university, the teaching innovation projects are evaluated and approved by this institutional review board-. Furthermore, it was endorsed by the Faculty of Nursing and Physiotherapy at the University of the Balearic Islands. All the students who took part were supplied with the information required about the activities involved, and were invited to request more information if they considered it necessary. The introductory text of the online questionnaire reminded the students of the voluntary nature of this activity, explaining that a teaching innovation project was being carried out and that its performance or non-performance would have no bearing on the students’ grades for the subject. The questionnaires did not include personal data that would allow the identification of the participants. Therefore, the participants’ consent was implicit in their choosing to take part. The results of the SBA were anonymised.

## Results

The Pre-S questionnaire was completed by 73 students, 41% of those enrolled in the subject. Their sociodemographic characteristics are listed in Table 3. The SBA formed part of the class subject evaluation activities, which were performed by all 179 enrolled students (12% male; 88% female). The open question was answered by 42 students (23.5%).

**Table 3:** Respondents’ characteristics

*n* = 73		
**Gender**		
Male	8	11%
Female	65	89%
**Age (years)**		
18–25	44	60.3%
26–39	12	16.4%
> 40	9	12.3%
Not recorded	8	11%
**Health work experience**		
Yes	22	30.2%
No	43	58.9%
Not recorded	8	10.9%
**Subject repeaters**		
Yes	10	13.7%
No	56	76.7%
Not recorded	7	9.6%

The following section describes the results obtained by the students’ competency in safe medication administration and their perceptions of the SBA.

### Students’ competency in safe medication administration: applying the six rights

**Fig. 1:**
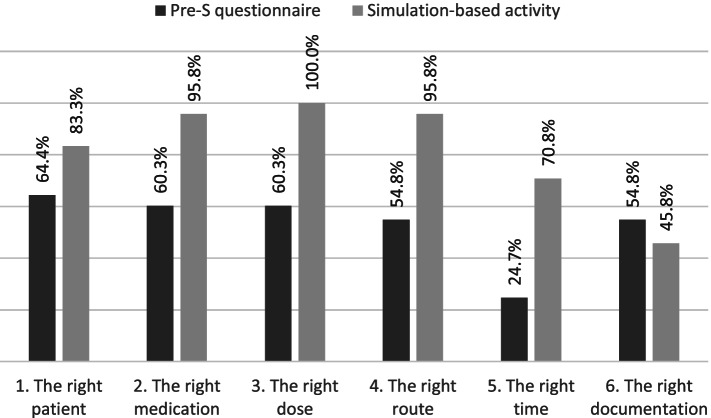
Results obtained in the Pre-S questionnaire and the SBA

The results obtained in the Pre-S questionnaire and the subsequent SBA are shown in Fig. 1.

In the Pre-S questionnaire, 64.4% of the students described alternative means of correctly identifying the patient. The action most frequently proposed was to ask the patient directly, while verification by reference to the patient’s identification bracelet was mentioned infrequently. A surprising fact was that some students proposed identifying patients according to their symptoms, an approach that is invalid for this purpose. The students' ability to identify the patient in situ improved during the SBA, when 83.3% performed this task appropriately, whether by checking the patient's room number, bed and name and/or by inspecting the identity bracelet.

In the Pre-S questionnaire, the majority of students (87.7%) correctly recognised at least one of the two drugs and 65.8% correctly identified the drug that could provoke an anaphylactic reaction. However, in response to an open question, the students had greater difficulty in describing all the activities that should be performed immediately before preparing the medication for administration. The aspects that were most commonly mentioned were checking the expiration date and the condition of the drug packaging (for example: the package is not deteriorated nor damaged); while checking the name of the drug in the presentation available and checking the integrity of pharmaceutical form itself (for example: the injectable solution was not crystallized) were less frequently mentioned. The verification of the correct medication improved during the SBA, with correct results being obtained in 95.8% of the cases. The teacher supervising this activity performed an in-situ evaluation of how well the students selected the correct drug, distinguished it from others with a similar appearance and verified the condition of the packaging and of the medication.

In their answers to the Pre-S questionnaire, 60.3% of the students calculated the dose correctly. During the simulation, all the groups correctly calculated the dose (reflecting the fact that the time spent preparing the activity was mainly dedicated to this calculation).

In the Pre-S questionnaire, 54.8% of the students correctly stated the correct route of administration for the proposed drug. The information about the route of administration of the drug was omitted intentionally from the case files of the SBA—the purpose of this omission was to verify that the students explicitly requested it. During the activity, only one group requested this information from the teacher.

In relation to the right time, the Pre-S questionnaire presented a work overload situation in which the student had to prioritise the administration of two drugs to two different patients. The results of this exercise were unsatisfactory, with only 24.7% of the students correctly assessing the situation. During the SBA, these results improved to 70.8%. The students who failed to observe the right time principle explained during the debriefing that this was because they did not take the question of time into account.

The right documentation in the Pre-S questionnaire was satisfactorily performed by 54.8% of the students. The dose was correctly recorded, but most of the students failed to evaluate its effectiveness. In the SBA, the data documentation had to be performed spontaneously by the students, and only 45.8% did so.

### Students’ perceptions of the SBA

Two categories emerged from the analysis of the answers given to the open question on perceptions of the SBA, related to the positive and negative aspects of the activity.

In relation to the positive aspects of the activity, the students expressed their satisfaction with the simulation experience. They consider the SBA a dynamic, agreeable and enjouable activity:

*“I found the seminars interesting because we were able to put into practice things that in class might not have been properly understood.”*

*“What I liked most was the way in which the seminars were given. They were fun.”*

Furthermore, the students stated the usefulness of simulation in their future professional activity because it approximates them to the reality of patient care:

*“The seminars were very interesting and, above all, extremely useful.”*

*“I found the seminars very useful because they placed us in real-life situations.”*

The students consider the simulation is a good way to improve dose calculation:

*“This year, medication dose calculation was explained in a way that was very good for us.”*

*“What I found most interesting was to practice dose calculation, since that’s what I have most trouble with.”*

Finally, with the SBA, the students became aware of the importance of the teamwork and their responsibility during drug administration:

*“In our profession, teamwork is important.”*

*“Acquiring a general idea of what pharmacology is about, since it’s a very important part of our profession and one of great responsibility.”*

Secondly, the students refer to some negative or improvement aspects in relation to the implementation of the simulation. Regarding areas in which the experience could be improved, the students suggest that the simulations should be conducted with smaller numbers of students and more time for simulated activities is needed:

*“The numbers in each group were quite large (…) If there were fewer people, I think there would be more interest in taking part.”*

*“There should have been more time to do this work or more time in the seminar.”*

A surprising finding was the students’ (mis)perception that learning and applying the six rights was restricted to the area of pharmacology:

*“With all the subjects we have to study, it’s impossible to absorb everything in just one term.”*

## Discussion

Our study shows that very positive results were obtained from using simulation as a means of teaching professional skills such as the safe administration of nursing medication, a finding that corroborates previous research in this field [18, 26–30, 43]. In general, better results were obtained for the six rights during the simulation than in the prior questionnaire. This can be explained because the Pre-S questionnaire was carried out individually, while the situation raised in the SBA was resolved from cooperative learning.

Our analysis of this teaching experience coincides with that of Shearer [32], who reported that the students’ identification of their patients improved substantially after the simulation exercise. However, although more than half of the students correctly identified the patient during the SBA, they did not systematically use the identification bracelet, which is the method normally recommended [10, 32, 40]. In this respect, our findings concur with those of previous research according to which patient identification error can occur in up to 80% of cases [32]. The question of patient identification was commonly raised in the debriefing sessions, when students realised the potential repercussions of such an error, like Avraham [7]. For example, if the patient had been allergic, this mistake could have caused anaphylactic shock and possibly death.

As in other studies [7, 39], notable improvements were observed in terms of achieving the right drug, the right dose, the right route and the right time during the simulation, compared with the situation recorded in the Pre-S questionnaire. Specifically, the students reported that simulation helped them learn dose calculation, which is a major initial source of stress and concern. In this respect, our results coincide with those of Harris et al. [26] and Dutra [44]. However, in the simulation situation the dose calculation was performed by the group as a whole, and not individually as in the Pre-S questionnaire. As observed in previous research [7, 28], this competency should be assessed in the individual, not the group, as difficulties might not otherwise become apparent.

The right time was the most difficult point to assess in both tests. As explained above, in the questionnaire the students were asked to resolve a situation of work overload and to perform the necessary prioritisation of tasks. This activity may have been complicated by their still incipient knowledge of pharmacology, lack of clinical reasoning skills and continuing need to develop their overall view of the patient and of the clinical unit [11, 31]. Furthermore, they did not ask “what time it was”. On the other hand, we should acknowledge the real difficulty faced by the student in practising a task of this type within a simulated situation. Having a single prescription leads students to assume that this is the medication to administer during the simulation without verifying that the simulated time is the time the drug should be administered. As an improvement strategy for the verification of this right, we could propose that the student have a 24-h prescription of drugs. The 24-h prescription of drugs would force students to check more explicitly the medications to administer at a given time (right time). Furthermore, it’s a way to improve the scenario reliabity.

The only task that was better performed in the questionnaire than in the subsequent simulation was that of the right documentation. This may be because the students were asked directly about the content of the record made, and therefore it was always present as an activity to be performed. It is difficult to compare this principle with its performance in the simulation, because although the first five of the rights arose spontaneously and were necessary in order to simulate the administration of medication, the documentation process was overlooked in most cases. The students’ failure to perform the necessary data documentation shows that while they had mostly assimilated the activities aimed at preventing medication-related errors, they did not realise that the process also involves recording the medication administered and controlling its subsequent effects. These results highlight the need to integrate this action into checklists for safe medication administration, as indicated in previous studies [4, 40].

In line with the INACSL Standards Committee [38, 45] and other research [7, 27, 28], we found debriefing to be of crucial importance to the learning process. This aspect of the SBA facilitated the subsequent application of patient safety culture and provided a space for analysis of the errors committed, highlighting their causes and offering solutions to ensure they were not repeated. The significant advances achieved with the SBA are consistent with one of the principles of improving patient safety: the need to learn from adverse events [3]. The students were made aware of their responsibility to avoid medication errors, in line with Steiner [29], who observed that simulation allows students to address possible situations of shock without putting anyone – patients or students – in danger, thus protecting students from becoming second victims [46].

In relation to the second objective regarding the opinion of the students about the activity, the SBA was well received by the students, who appreciated the opportunity to have an initial contact with the reality of healthcare in a controlled environment and to learn relevant skills in an innovative way. Simulation enabled these students to better understand pharmacology concepts and to apply them in a realistic context. Similar opinions have been expressed in previous studies in this field [8, 21, 24, 28, 29].

In our study, the students highlighted the need for more time in which to perform the activity and for the groups to be smaller. In this, they seconded the recommendations of the INACSL [38]. This Teaching Innovation Project is part of the Faculty plan for the implementation of clinical simulation in nursing studies. It is expected that in the future these teaching sessions can be adapted to a greater extent to the simulation standards [45]. Indeed, some studies have examined one-on-one simulation exercises, designed to give the student the opportunity to complete all stages of the medication administration process alone, as is often the case in practice [7, 28]. However, it should be noted that appropriate resources need to be assigned to this type of teaching method [32] and the Nursing degree curriculum might need to be adjusted appropriately.

### Limitations

This study presents certain limitations, especially the difficulty encountered in comparing the results of project activities. Thus, the Pre-S questionnaire was evaluated individually, while in the SBA a group evaluation was performed. In addition, the techniques used in the two moments to evaluate each of the right ones were different. The lack of validated tools for assessing the competency of safe drug administration during the simulation has limited the generalization of our results. Moreover, the small number of students participating in this study and their recruitment from the same institution limits the generalisability of our findings to other populations and settings.

### Implications

First, despite the methodological limitations identified, we consider that the results are of interest to the nursing teaching community, given the low number of studies related to simulation as a methodology for teaching safe medication administration in the Spanish context. The incorporation of this teaching methodologies raises the need to make efforts in the teaching curriculum in order to increase its use during the nursing degree. For optimum effectiveness, further resources are needed for simulation, such as appropriate spaces, teachers trained in this methodology, more time for the preparation and design of the scenarios and smaller groups and/or individual evaluation. Second, the development of new teaching methodologies must be accompanied by evaluations through different research designs despite de difficulties of investigating within the classroom and randomizing research without discriminating against students in their learning process [44]. Third, the improvement of competence in the safe medication administration by students will result in clinical practice, improving patient safety and protecting students from becoming second victims.

## Conclusions

As a teaching method, simulation is shown to be a useful means of acquiring competence in the safe administration of medication. In this sense, strategies should be intensified to ensure correct patient identification and the correct documentation of the procedures followed. However, these methodologies must be developed in order to be able to individually evaluate this acquisition.

On the other hand, the students are satisfied that the simulation bridged the gap between theory and practice and brought them closer to the realities of the healthcare system. Students perceive that the simulation methodology is more appropriate than lectures or other conventional theoretical activities. The general opinion of the students confirms that the SBA increases the understanding of the process of administering medication and the awareness of their professional responsibility during this process.

## Data Availability

The datasets generated and/or analysed during the current study are not publicly available due to privacy and confidentiality reasons, but are available from the corresponding author on reasonable request.

## Abbreviations

INACSL: International Nursing Association for Clinical Simulation and Learning
MASAT: Medication Administration Safety Assessment Tool
Pre-S questionnaire: Pre-Simulation questionnaire
SBA: Simulation-based activity
WHO: World Health Organization
