# Correction: Use of simulation to improve nursing students’ medication administration competence: a mixed-method study

**DOI:** 10.1186/s12912-022-00920-3

**Published:** 2022-06-14

**Authors:** Sandra Pol-Castañeda, Alba Carrero-Planells, Cristina Moreno-Mulet

**Affiliations:** 1 Department of Nursing and Physiotherapy, University of the Balearic Islands, Palma, Balearic Islands Spain; 2 Care, Chronicity and Health Evidences Research Group, Health Research Institute of the Balearic Islands (IdISBa), 07010 Palma, Spain

**Correction: BMC Nurs 21, 117 (2022)**

**https://doi.org/10.1186/s12912-022-00897-z**

Following publication of the original article [1], the authors reported an error in the given name and family name designation, the given name and family name need to be interchanged.

The correct author names have been provided in this Correction.

The original article [1] has been corrected.
